# Nonalcoholic fatty liver disease and risk of incident hypertension: a systematic review and meta-analysis

**DOI:** 10.1097/MEG.0000000000002299

**Published:** 2021-11-03

**Authors:** Stefano Ciardullo, Guido Grassi, Giuseppe Mancia, Gianluca Perseghin

**Affiliations:** a Department of Medicine and Rehabilitation, Policlinico di Monza Hospital, Monza; b Department of Medicine and Surgery, School of Medicine and Surgery; c Department of Medicine and Surgery, Clinica medica, University of Milano Bicocca, Milan, Italy

**Keywords:** hypertension, incidence, Nonalcoholic fatty liver disease, ultrasound

## Abstract

Several studies reported an association between nonalcoholic fatty liver disease (NAFLD) and the risk of incident hypertension. The objective of this systematic review and meta-analysis was to obtain a precise and reliable estimate of the nature and magnitude of this association. We systematically searched *Ovid*-*MEDLINE* up to March 2021 for observational studies in which NAFLD was diagnosed in adults using blood-based panels, imaging techniques or liver biopsy and with a follow-up ≥1 year. Measures of association from individual studies were meta-analyzed using random-effects models. Of the 1108 titles initially scrutinized, we included 11 cohort studies with data on 390 348 participants (52% male) and a mean follow-up of 5.7 years. In the overall analysis, NAFLD was associated with a moderately increased risk of incident hypertension (hazard ratio 1.66; 95% confidence interval (CI), 1.38-2.01; test for overall effect z = 5.266; *P* < 0.001). There was significant heterogeneity among the studies (*P* < 0.001). Sensitivity analyses showed that estimates were not affected by geographical location, duration of follow-up and adjustment for baseline blood pressure values. On the other hand, the magnitude of the association was lower in studies that adjusted for baseline adiposity compared with those that did not, explaining part of the observed heterogeneity. No significant publication bias was detected by funnel plot analysis and Egger’s and Begg’s tests. This large meta-analysis indicates that NAFLD is associated with a ~1.6-fold increased risk of developing hypertension. Further studies are needed to investigate the role of NAFLD severity in terms of inflammation and fibrosis on incident hypertension.

## Abstract 2

Supplemental Digital Content is available in the text.

## Introduction

Nonalcoholic fatty liver disease (NAFLD) is the most common form of chronic liver disease, affecting ~25% of the adult world population [1]. It is an umbrella term including patients with different degrees of histologic severity spanning from simple steatosis to lobular inflammation and hepatocyte ballooning (nonalcoholic steatohepatitis) to collagen deposition leading to liver fibrosis and possibly cirrhosis [2,3]. It is increasingly recognized as a frequent cause of liver-related morbidity and mortality and its global prevalence is expected to further increase in the foreseeable future, given the widespread rise in obesity rates among adolescents and young adults [4].

Being frequently associated with insulin resistance and ectopic fat deposition, its prevalence is even higher in patients with metabolic disorders, such as type 2 diabetes [5], and in patients displaying signs of the metabolic syndrome, including visceral obesity, dyslipidemia and essential hypertension [6–8].

Accumulating evidence suggests that NAFLD is associated with an increased prevalence and incidence of hypertension [9,10], which still represents by far the most common disease that affects human beings and is considered the top contributor to the burden of disease worldwide [11–13].

To date, two previous meta-analyses examined the association between γ-glutamyl transpeptidase (γ-GT) levels and risk of incident hypertension [14,15], whereas no quantitative summary of the available evidence is present on studies using more accurate measures of liver fat content such as specific blood-based panels, imaging techniques or liver biopsy.

We have therefore undertaken a systematic review and meta-analysis of observational cohort studies of adults from different geographical locations examining the association between NAFLD (diagnosed based on imaging, blood biomarkers or liver biopsy) and incident hypertension. A meta-analytic approach might help resolve inconsistencies among previously published studies and more precisely define the nature and the magnitude of the association.

## Methods

The data of the meta-analysis are available from the corresponding author at reasonable request.

### Data sources and search strategy

We systematically searched *Ovid*-*MEDLINE* to identify articles reporting the results of longitudinal observational studies published up to March 2021 investigating the association between NAFLD and incident hypertension. The search, designed by S.C. and G.P., was performed by S.C. Articles were selected by using the terms “nonalcoholic fatty liver disease” OR “NAFLD” OR “fatty liver” OR “nonalcoholic steatohepatitis” AND “incidence” OR “new-onset” AND “hypertension” (Supplementary Table S1, Supplemental digital content 1, http://links.lww.com/EJGH/A725). We limited our searches to human studies without predefined language restrictions. Reference lists of included manuscripts and review articles were hand searched to identify additional studies not covered by the original database searches. The systematic review was performed in accordance with the Preferred Reporting Items for Systematic Reviews and Meta-Analysis (PRISMA) as outlined in Supplementary Table S2, Supplemental digital content 1, http://links.lww.com/EJGH/A72516. Given the observational nature of the included studies, we followed the reporting items proposed by the Meta-analysis Of Observational Studies in Epidemiology for the meta-analysis of these studies [16].

### Study selection

Only studies that met the following inclusion criteria were considered for the present systematic review and meta-analysis: (1) longitudinal design; (2) duration of follow-up ≥1 year; (3) assessment of the relationship between NAFLD and incident hypertension; (4) availability of a measure of association [hazard ratio or odds ratio (OR)] with 95% confidence intervals (CI) for the outcome of interest; (5) a diagnosis of liver steatosis based either on imaging techniques (ultrasonography, computerized tomography or transient elastography), blood/biomarkers [fatty liver index (FLI) [17], hepatic steatosis index [18] or other scores of liver steatosis] or liver biopsy and (6) a diagnosis of hypertension based on office blood pressure (BP) measurement by physicians or International Classification of Diseases (ICD) codes. Exclusion criteria were as follows: (1) cross-sectional studies, editorials, congress abstracts and case reports; (2) studies that did not exclude different causes of liver steatosis; (3) studies with a median follow-up <1 year; (4) studies that did not report a measure of association with 95% CI for the outcome of interest and (5) studies that were performed in the pediatric population.

### Data extraction and quality assessment

All titles and abstracts were independently examined by two investigators (S.C. and G.P.) and full-texts of potentially relevant articles were obtained and scrutinized separately by the same authors. We resolved discrepancies by consensus, referring back to the original articles. Information was extracted from all studies on study design, country, follow-up duration, the outcome of interest and covariates included in the multivariable regression models. In case of multiple publications on the same subjects, we included only the most up-to-date and comprehensive one. The risk of bias was assessed independently by two authors (S.C. and G.P.) and discrepancies were resolved by discussion. Studies were evaluated for their quality following the Newcastle-Ottawa Scale (NOS) [19]. This scale allocates a maximum of nine points for three major domains: selection of participants (maximum of four points), comparability of study groups (maximum of two points) and ascertainment of outcomes of interest (maximum of three points).

### Data synthesis and statistical analysis

Hazard ratios or ORs and corresponding 95% CI were considered as the measure of association of interest for each eligible study. We extracted the effect size from the statistical model reporting the maximum extent of adjustment for confounders. Adjusted hazard ratios and OR were pooled to calculate an overall estimate of effect size. Because we expected a relatively large heterogeneity in results, as it is a common finding when evaluating observational studies on different cohorts with varying degrees of adjustment, we used the random-effects model using the method of Der Simonian and Laird, with the estimate of heterogeneity being taken from the Mantel–Haenszel model. Statistical heterogeneity was evaluated by visual inspection of the forest plot, as well as by the Cochrane Q test and the *I*^2^ statistics, which represents the proportion of the observed variability that cannot be explained by chance alone.

A funnel plot was constructed to evaluate the presence of publication bias by plotting the logarithm of the effect measure against the logarithm of its standard error. We also used both the Egger’s test [20] and the rank correlation Begg’s test [21]. To evaluate the possible sources of heterogeneity and the robustness of our findings, we performed prespecified subgroup-sensitivity analyses by geographical location, methodology used to diagnose NAFLD and degree of covariate adjustment (with special focus on adjustment for baseline BP values and measures of adiposity). Moreover, additional sensitivity analyses were conducted to evaluate whether the pooled effect estimate was strongly influenced by a specific study. This was performed by omitting one study each time and recalculating the pooled effect estimate on the remaining studies. All statistical analyses were performed with Stata 13.0 (Stata Corp, College Station, Texas, USA). A two-tailed *P* value <0.05 was considered significant.

## Results

### Search results

**Fig. 1.**
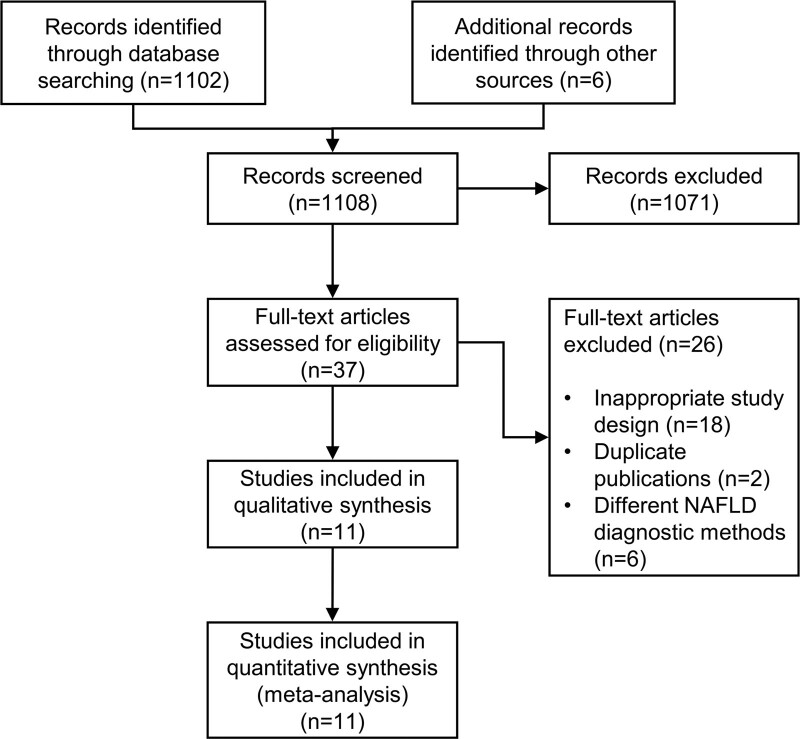
PRISMA flow diagram. PRISMA, Preferred Reporting Items for Systematic Reviews and Meta-Analyses.

From a total of 1108 articles identified by literature research, 1071 were excluded by title and abstract screening. We examined the full text of the remaining 37 studies. After excluding articles with a cross-sectional design or that did not report the outcome of interest (*n* = 18), 2 studies were not included because they reported results on the same population of two included studies, and 6 were excluded because they used different diagnostic methods to define NAFLD (mainly γ-GT levels), leading to a final number of 11 included studies that were analyzed and assessed for quality. A PRISMA flow diagram of the study selection is shown in Fig. 1.

### Features of the included articles

The main characteristics of the included studies are reported in Table 1. All were observational (either prospective or retrospective) cohort studies and most of them were performed on middle-aged individuals sampled from the general population. Overall, they included 390 348 individuals (52% men) with a mean follow-up of 5.7 years (ranging from 2.6 to 9 years). Eight studies were carried out in Asia (South Korea and China), two in Europe (France and Germany) and one in the USA. Excluding one study that did not report the prevalence of NAFLD but segregated the population in FLI quartiles [29], the mean prevalence of NAFLD was 21.5%. One study was performed only in men, while all the rest included a combined sample of men and women.

**Table 1.** Overview of the included studies investigating the association between nonalcoholic fatty liver disease and incident hypertension

Author	Year	Country	Follow-up (years)	Sample	Male (%)	NAFLD diagnostic method	NAFLD at baseline (%)	Diabetes at baseline (%)	Definition of hypertension	Adjustment
Bonn*et et al.*, [22]	2017	France	9	2886	45.2	Fatty liver index	7.6	NA	BP ≥140/90 mmHg or use of BP lowering drugs	Age, sex, smoking, FPG and alcohol intake
Fan *et al*.,[23]	2007	China	6	1146	90.5	Ultrasound	31.2	6.5	BP ≥140/90 mmHg	Age
Huh *et al*.,[24]	2015	South Korea	2.6	1521	31.8	Fatty liver index	8.2	NA	BP ≥140/90 mmHg or use of BP lowering drugs	Age, sex, SBP, DBP, smoke, exercise, alcohol, diabetes
Kim *et al*.,[25]	2017	South Korea	5.1	2119	54.1	Ultrasound	19.8	2.8	BP ≥140/90 mmHg or use of BP lowering drugs	Age, sex, smoking, waist circumference, triglycerides, HDL, LDL, uric acid
Lau *et al*.,[26]	2010	Germany	5	2417	63.4	Ultrasound	39.4	7.2	BP ≥140/90 mmHg or use of BP lowering drugs	Age, sex, waist circumference
Liu *et al*.,[27]	2018	China	5	6704	36.3	Ultrasound	30	11.1	BP ≥140/90 mmHg or use of BP lowering drugs or self-reported diagnosis	Age, sex, smoking, alcohol, physical activity, education, family history, SBP, waist circumference, change in BMI
Ma *et al*.,[28]	2016	USA	6.2	1051	54.1	CT	17.8	2.6	BP ≥140/90 mmHg or use of BP lowering drugs	Age, sex, smoking, physical activity, alcohol intake, SBP, DBP, BMI, change in BMI
Roh *et al*.,[29]	2020	South Korea	5.2	334280	48.3	Fatty liver index	NA	0.0	ICD-10 code	Age, sex, alcohol, SBP, DBP, glucose, total cholesterol
Ryoo *et al*.,[30]	2014	South Korea	5	22090	100	Ultrasound	34.2	2.8	BP ≥140/90 mmHg or use of BP lowering drugs	Age, BMI, triglyceride, creatinine, transaminases, smoking, exercise, diabetes
Sung *et al*.,[31]	2014	South Korea	5	11448	69.4	Ultrasound	19.9	2.1	BP ≥140/90 mmHg or use of BP lowering drugs	Age, sex, smoking, alcohol, exercise, SBP, BMI, diabetes, GGT, HOMA-IR, eGFR, change in BMI
Zhou and Cen [32]	2018	China	9	4686	67.8	Fatty liver index	6.5	NA	BP ≥140/90 mmHg or use of BP lowering drugs	Age, sex, waist circumference, SBP, DBP, FPG, HDL-C, TG

BP, blood pressure; eGFR, estimated glomerular filtration rate; FPG, fasting plasma glucose; GGT, gamma-glutamyl transpeptidase; HDL-C, high density lipoprotein cholesterol; HOMA-IR, homeostatic model of insulin resistance; ICD, International Classification of Diseases; LDL, low density lipoprotein; NA, not available; NAFLD, nonalcoholic fatty liver disease; TG, triglycerides.

Six studies used ultrasonography to diagnose NAFLD (*n* = 45 924 individuals), one study used computed tomography (*n* = 1051 individuals) and the remaining four used the FLI (*n* = 343 373). Definition of hypertension was consistent in most studies and as BP ≥140/90 mmHg or the initiation of antihypertensive treatment, with one study identifying patients using ICD codes. As reported in Supplementary Table S3, Supplemental digital content 1, http://links.lww.com/EJGH/A725 4, 6 and 1, studies were considered at low (receiving at least 8 stars), medium (7 stars) and high risk of bias (<7 stars) according to NOS, respectively, thus indicating an overall low to medium risk of bias.

### Association between nonalcoholic fatty liver disease and incident hypertension

**Fig. 2.**
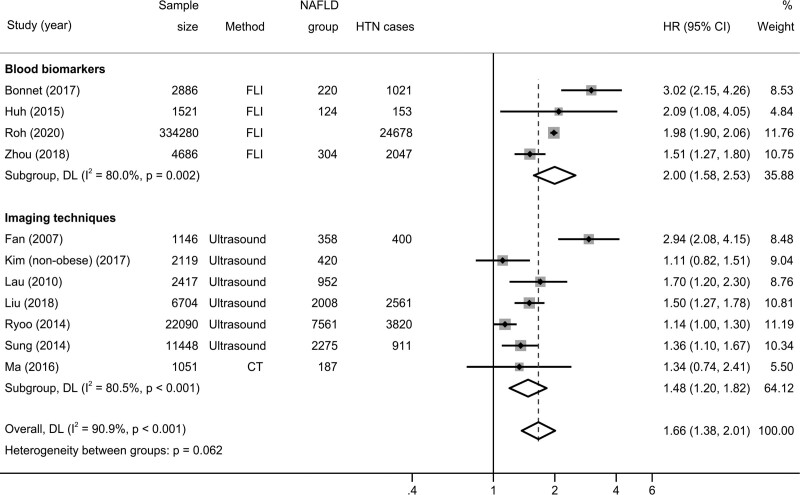
Forest plot and pooled estimates on the effect of NAFLD on the risk of incident hypertension in 11 eligible studies, stratified based on the methodology used for NAFLD diagnosis. CI, confidence interval; HR, hazard ratio; HTN, hypertension; NAFLD, nonalcoholic fatty liver disease.

As shown in Fig. 2, the pooled hazard ratios for incident hypertension was 1.66 (95% CI, 1.38–2.01; test for overall effect *z* = 5.266; *P* < 0.001) when pooling adjusted effect estimates. The test for heterogeneity was significant (Cochran’s Q = 109.85; degrees of freedom (df) = 10; *P* < 0.001). No study suggested a decreased risk of incident hypertension in patients with NAFLD.

When the analysis was stratified based on the methodology used to identify patients with NAFLD, the association of interest was consistent in both studies using FLI (*n* = 4 studies; hazard ratios 2.00; 95% CI, 1.58–2.53; test for overall effect *z* = 5.766; *P* < 0.001) and studies using imaging techniques such as ultrasonography or CT (*n* = 7 studies; hazard ratios 1.48; 95% CI, 1.20–1.82; test for overall effect *z* = 3.657; *P* < 0.001), with borderline heterogeneity between the two groups (Cochran’s Q = 3.49; degrees of freedom (df) = 1; *P* = 0.062).

### Sensitivity analyses and risk of publication bias

**Fig. 3.**
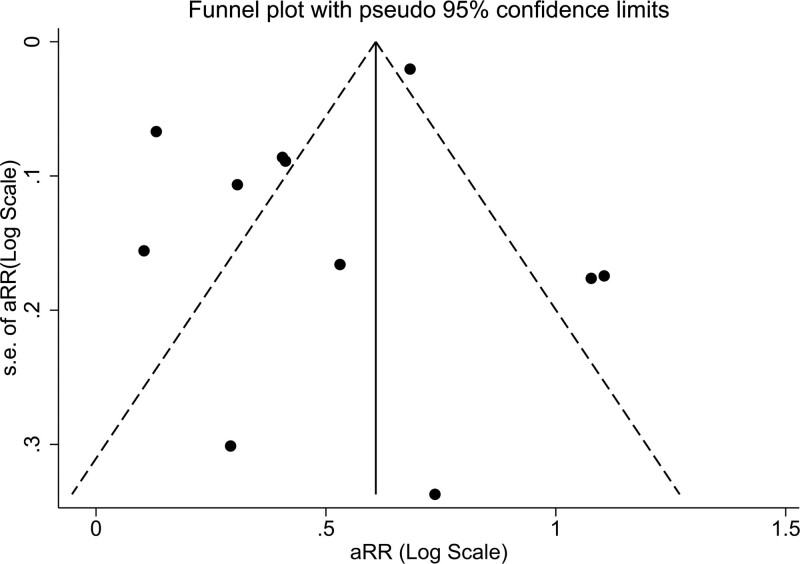
Funnel plot of selected studies describing the relationship between effect size and standard error on the log scale. The vertical line represents the pooled effect size and the dashed lines represent the pseudo 95% confidence intervals.

Subgroup analyses based on follow-up duration, degree of adjustment for covariates and geographical region were performed to explore possible sources of heterogeneity and are shown in Table 2. Notably, an increased risk of incident hypertension in patients with NAFLD was evident in all subgroups. No significant impact was found with regards to the duration of follow-up, geographical region and adjustment for baseline BP values. On the other hand, we found that adjustment for adiposity measure at baseline (either BMI or waist circumference or both) attenuated the extent of the association. Indeed, the hazard ratio was 2.44 (95% CI, 1.84–3.22; test for overall effect *z* = 6.229; *P* < 0.001) for those not performing the adjustment (*n* = 4 studies) and 1.36 (95% CI, 1.20–1.54; test for overall effect *z* = 4.871; *P* < 0.001) for those performing it (*n* = 7 studies), with a significant between-group heterogeneity in the outcome measure (Cochrane Q = 14; df = 1; *P* < 0.001). No evidence of significant publication bias was found by using asymmetry analysis in the funnel plot (Fig. 3). Furthermore, both Egger’s test (*P* = 0.247) and rank correlation Begg’s test (*P* = 0.312) did not show statistically significant asymmetry. Finally, sensitivity analysis (Supplementary Figure S1, Supplemental digital content 1, http://links.lww.com/EJGH/A725) showed that there was no significant trend suggesting that the overall result was influenced by any individual study.

**Table 2:** Subgroup-sensitivity analyses on studies investigating the association between nonalcoholic fatty liver disease and incident hypertension

	Hazard ratios (95% CI)	Test for overall effect	Study number	Between group heterogeneity
Duration of follow-up				
<6 years	1.48 (1.16–1.89)	*z* = 3.182, *P* = 0.001	7	*P* = 0.176
≥6 years	2.10 (1.35–3.20)	*z* = 3.325, *P* = 0.001	4	
Adjustment for baseline BP				
Absent	1.78 (1.16–2.73)	*z* = 2.647, *P* = 0.008	5	*P* = 0.655
Present	1.60 (1.33–1.93)	*z* = 4.881, *P* < 0.001	6	
Adjustment for adiposity^a^				
Absent	2.44 (1.84–3.22)	*z* = 6.229, *P* < 0.001	4	*P* < 0.001
Present	1.36 (1.20–1.54)	*z* = 4.871, *P* < 0.001	7	
Geographical region				
Europe/USA	1.97 (1.23–3.15)	*z* = 2.830, *P* = 0.005	3	*P* = 0.401
Asia	1.58 (1.27–1.96)	*z* = 4.078, *P* < 0.001	8	

All studies included in Fig. 2 were analyzed in these subgroup analyses.

a Inclusion of either BMI or waist circumference in the multivariable logistic regression model.

BP, blood pressure; CI, confidence interval.

## Discussion

In this large meta-analysis including 11 observational cohort studies involving 390 348 adult individuals free from hypertension at baseline from different geographical locations, we show that NAFLD is associated with a hazard ratio of 1.66 (95% CI, 1.38–2.01) for incident hypertension over a mean follow-up of 5.7 years. The extent of the association did not differ when the analysis was stratified based on diagnostic modality (blood-based panels versus imaging techniques), country of origin and adjustment for baseline BP values. On the other hand, the hazard ratio from studies that adjusted their estimates for adiposity measures (waist circumference and BMI) at baseline or at follow-up was significantly lower than that of studies that did not perform this correction, even though the association remained significant. This aspect underlies the important role of obesity as a potential confounder.

The results of the present study expand those of two previous meta-analyses focusing on the role of γ-GT as a predictor of incident hypertension [14,15]. The most recent, by Kunutsor *et al*., [14] which included 14 studies for a total of 44 582 individuals, found that in a comparison of extreme thirds of baseline γ-GT levels, the relative risk for hypertension was 1.32 (95% CI, 1.23–1.43), with the heterogeneity of estimates from different studies being explained by mean age, duration of follow-up and degree of confounder adjustment. Compared to these results, we have significantly increased the sample size (about nine times) and identified NAFLD with more accurate diagnostic methods, as γ-GT levels might be affected by a series of unrelated conditions such as alcoholic liver disease, cholestatic liver disease and induction by drugs [33]. On the other hand, we cannot provide evidence on whether NAFLD severity in terms of inflammation and fibrosis impacts the magnitude of this association, as was recently suggested in a biopsy-based study involving patients with NAFLD and different degrees of histologic changes [34]. On this aspect, additional cohort studies of well-characterized NAFLD patients are needed.

From a pathophysiological standpoint, several mechanisms might account for the role of NAFLD as a potential driver of hypertension in the general population [6]. It is well known that liver steatosis is strongly associated with insulin resistance and hyperinsulinemia. Apart from increasing the risk of developing type 2 diabetes, insulin resistance is associated with low-grade systemic inflammation and endothelial dysfunction, which might lead to vasoconstriction. Moreover, the action of insulin on sodium handling is frequently preserved in insulin resistance and contributes to sodium retention and arterial hypertension [35]. Other pathways linking the two conditions are represented by oxidative stress, hyperactivity of the sympathetic nervous system and the angiotensin aldosterone systems as well as increased risk of chronic kidney disease [36].

The current meta-analysis has several limitations that deserve to be acknowledged. First, given the observational nature of the included studies, it is not possible to definitely prove a causality link between the exposure and the outcome. Second, while most studies adjusted for several potential confounders including age, cigarette smoke and baseline BP values (as shown in Table 1), the possibility of residual confounding by unmeasured factors cannot be excluded. As an example, some studies did not adjust for baseline BMI and waist circumference. It should be noted, however, that these parameters are included in the FLI equation and adjustment might therefore reduce the diagnostic ability of the score to correctly identify patients with steatosis and therefore bias results towards the null. It was therefore not possible to combine models that accounted for the same variables. While sensitivity analyses showed consistency of the association of interest independently of geographical region, most studies included Asian patients, who tend to develop NAFLD at lower BMI levels compared to patients of Caucasian origin and this aspect may influence the observed effect of adiposity in modulating the relationship between NAFLD and hypertension.

Third, interpretation of our results demands cautiousness given the high degree of heterogeneity found between studies. While no study found a lower risk of hypertension in patients with NAFLD, variability in the magnitude of the association might result from a combination of factors including covariate adjustment, methods for NAFLD diagnosis and other potential unmeasured variables. It should also be noted that thresholds for significant alcohol consumption differed among the included studies, and not all of them systematically screened all participants for different forms of liver disease and use of steatogenic medications. More detailed analysis of heterogeneity would require pooling individual participants’ data from the different studies.

Fourth, none of the included studies used a gold standard technique such as liver biopsy or magnetic resonance spectroscopy to diagnose NAFLD. In fact, while these two techniques are more reliable than both liver ultrasonography and FLI, they are expensive and time-consuming, making them unsuitable for large-scale population studies. Moreover, liver biopsy is an invasive technique with possible (although rare) life-threatening complications, raising ethical concerns related to its use in apparently healthy subjects [37,38].

Our analysis also has some important strengths. It incorporates data from large epidemiological studies from Asia, Europe and the US including a representative pool of patients with NAFLD seen in clinical practice. Moreover, the large number of both exposed individuals and events yields high statistical power to precisely quantify the association between NAFLD and incident hypertension. Finally, there was no sign of significant publication bias affecting the analysis when evaluated by both Egger’s and Begg’s tests.

In conclusion, this large meta-analysis shows that NAFLD (diagnosed by either FLI or imaging techniques) is significantly associated with a ~1.7-fold increased risk of developing hypertension over a mean of 5.7 years. Moreover, obesity is an important confounder responsible for significant heterogeneity between studies and affecting the extent of this association. This underlies the need to carefully screen patients with NAFLD for the development of hypertension and the associated risk of cardiovascular events. Further studies evaluating whether NAFLD severity in terms of inflammation and fibrosis impacts on the risk of developing hypertension are needed.

## Acknowledgements

All authors made substantial contributions to the conception and design or acquisition, analysis and interpretation of data. All authors drafted the article or revised it critically for important intellectual content. All authors approved the final version of the manuscript to be published. GP is the guarantor of this work.

### Conflicts of interest

There are no conflicts of interest.

## Supplementary Material


